# Evidence map of studies evaluating methods for conducting, interpreting and reporting overviews of systematic reviews of interventions: rationale and design

**DOI:** 10.1186/s13643-015-0178-0

**Published:** 2016-01-06

**Authors:** Carole Lunny, Sue E. Brennan, Steve McDonald, Joanne E. McKenzie

**Affiliations:** Australasian Cochrane Centre, School of Public Health and Preventive Medicine, Monash University, 549 St Kilda Road, Melbourne, VIC 3004 Australia

**Keywords:** Evidence map, Overviews of systematic reviews, Evidenced-based methods

## Abstract

**Background:**

Overviews of systematic reviews attempt to systematically retrieve and summarise the results of multiple systematic reviews into a single document. Methods for conducting, interpreting and reporting overviews of reviews are in their infancy. To date, there has been no systematic review or evidence map examining the range of methods for overviews nor of the evidence for using these methods. The objectives of the study are to develop and populate a framework of methods that have or may be used in conducting, interpreting and reporting overviews of systematic reviews of interventions (stage I); create an evidence map of studies that have evaluated these methods (stage II); and identify and describe unique methodological challenges of overviews.

**Methods:**

The research will be undertaken in two stages. For both stages, we plan to search methods collections (e.g. Cochrane Methodology Register, Meth4ReSyn library, AHRQ Effective Health Care Program) to identify eligible studies. These searches will be supplemented by searching reference lists and citation searching. Stage I: Methods used in overviews will be identified from articles describing methods for overviews, methods studies examining a cross section/cohort of overviews, guidance documents and commentaries. The identified methods will populate a framework of available methods for conducting an overview. Two reviewers will independently code included studies to develop the framework. Thematic analysis of the coded data will be used to categorise and describe methods. Stage II: Evaluations of the performance of methods will be identified from systematic reviews of methods studies and methods studies. Evaluations will be described and mapped to the framework of methods identified in stage I.

**Discussion:**

The results of this process will be useful for mapping of methods for overviews of systematic reviews, informing guidance and identifying and prioritising method research in this field.

**Electronic supplementary material:**

The online version of this article (doi:10.1186/s13643-015-0178-0) contains supplementary material, which is available to authorized users.

## Background

Overviews of systematic reviews (or umbrella reviews) attempt to systematically retrieve and summarise the results of multiple systematic reviews into a single document [1]. The number of published overviews of systematic reviews (henceforth termed overviews) has increased steadily in recent years, in part due to the proliferation of systematic reviews [2]. Methods for conducting, interpreting and reporting overviews are in their infancy [2]. To date, there has been no systematic review or evidence map examining the range of methods for overviews (particularly those which are unique to overviews) nor of the evidence for using these methods.

In general, the steps for undertaking an overview mirror those of a systematic review, with many of the methods used in systematic reviews being directly transferrable to overviews (e.g. independent study selection and data extraction) [3]. However, there are unique features of overviews that require the use of different or additional methods, for example, methods for assessing the quality or the risk of bias in systematic reviews; dealing with the inclusion of the same trial in multiple systematic reviews; dealing with out-of-date systematic reviews; and dealing with discordant results across systematic reviews [2].

Evidence maps provide a systematic method for mapping the evidence on a particular topic, with the resulting map facilitating identification of gaps in the literature [4, 5]. Bragge [6] defines evidence mapping as describing the yield, design and characteristics of research in broad topic areas, in contrast to systematic reviews, which usually address narrowly focused research questions. Evidence mapping has been primarily used to map the evidence for healthcare interventions [7, 8]; however, the approach may also be usefully applied for mapping the evidence on other topics. For example, evidence mapping may be useful for collating evidence on the range and performance of research methods. To our knowledge, evidence mapping has yet to be applied in this way [5].

Where possible, methodological guidance for conducting overviews should be based on empirical research; that is, based on methods that have been evaluated and shown to have better performance [9]. It is therefore timely to systematically review and map the available methods literature to determine where there are gaps or areas of uncertainty and hence what methods research should be undertaken as a priority. We will apply the evidence mapping approach to studies evaluating the methods for conducting, interpreting and reporting overviews of systematic reviews of interventions.

The objectives of this study are to develop and populate a framework of methods that have been used, or may be used, in conducting, interpreting and reporting overviews of systematic reviews of interventions (stage I); (2) create an evidence map of studies that have evaluated these methods (stage II); and (3) identify and describe unique methodological challenges of overviews.

## Methods/design

The methods used in this study are based on published methods for mapping the evidence in broad content areas [4, 6, 10–13]. Methods for developing an evidence map involve several steps, namely, defining a topic and specific questions to be answered by the evidence map; searching for and selecting relevant studies; and reporting on the yield and study characteristics.

**Fig. 1:**
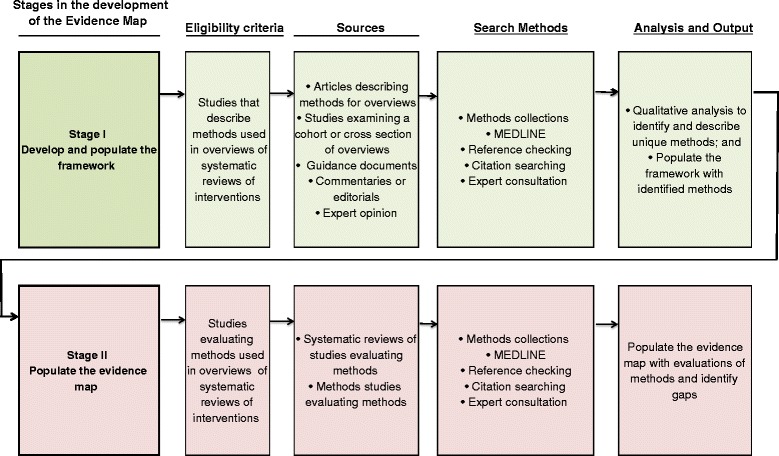
Stages in the development of an evidence map of overview methods

Our mapping study will involve two stages. In stage I, we will use the following sources to identify and describe methods that have been used or recommended for use in overviews: articles describing methods for overviews, methods studies that have described the characteristics of a cross section or cohort of overviews; guidance documents for conducting overviews; and commentaries or editorials. The identified methods will populate a framework of methods relating to the steps involved in conducting an overview (e.g. defining objectives, searching for systematic reviews, selecting studies). The second stage will involve the development of an evidence map of studies that have evaluated any of the identified methods (stage II). The steps involved in these stages are depicted in Fig. 1. The evidence map will facilitate identification of methods that have had little or no evaluation.

We have used the Preferred Reporting Items for Systematic reviews and Meta-Analyses Protocols (PRISMA-P) [14] to develop this protocol. The PRISMA-P checklist was developed for the preparation of protocols of systematic reviews that summarise aggregate data from studies, particularly the evaluations of the effects of interventions, and therefore, not all PRISMA-P items were applicable (Additional file 1). We did not register our evidence map on PROSPERO, the international prospective register of systematic reviews (http://www.crd.york.ac.uk/PROSPERO/), since reviews of ‘methodological issues’ do not met the inclusion criteria for this registry (as specified 29 June 2015).

### Search methods

Several sources will be searched to identify available methods (stage I) and studies that have evaluated the performance of those methods (stage II). We will undertake searches of the following methods collections: Cochrane Methodology Register, Meth4ReSyn library, the Scientific Resource Center Methods library of the AHRQ Effective Health Care Program, and Cochrane Colloquium abstracts. In addition, we will undertake a search of Ovid MEDLINE from 2000 onwards. We will use variations of the following search terms: overview, umbrella reviews, overviews of reviews, overviews of systematic reviews, meta-review or review of reviews (Additional file 2).

We will conduct purposive searches to locate evaluations of the methods we have identified, where the above searches are unlikely to have located these evaluations. For example, although searching for systematic reviews is a fundamental step of overviews, there are other purposes aside from overviews (e.g. developing guidelines) for which retrieving systematic reviews is necessary. Therefore, papers describing the development and evaluation of search strategies for systematic reviews may reasonably not have mentioned ‘overviews’ (or its synonyms) and thus would not be identified in the searches we describe above. Details of these additional search strategies will be documented and reported.

Given the potential difficulties in locating methods literature on overviews, we will supplement our searches by examining the reference lists of all included articles and undertaking citation searches of seminal papers using Google Scholar, Scopus and Web of Science. All three of these databases will be searched for citations because searches run in each database have been shown to return unique material [15]. In addition, as part of a related research project to develop a search strategy to identify overviews in MEDLINE, we set aside any methods papers that we identified through screening citations [16]. Finally, we will contact relevant experts in the field to help identify additional methods papers.

### Eligibility criteria

Separate eligibility criteria will be applied for stages I and II of the development of the evidence map (Fig. 1).

### Stage I: populating the framework—identification of methods used in overviews of systematic reviews of interventions

Inclusion criteria:

i) Articles describing methods for overviews
ii) Studies examining methods used in a cross section or cohort of overviews
iii) Guidance (e.g. handbooks and guidelines) for undertaking overviews
iv) Commentaries or editorials that discuss methods for overviews

Exclusion criteria:

i) Articles published in languages other than English
ii) Studies describing methods for network meta-analysis
iii) Articles exclusively about methods for overviews of other review types (i.e. not of interventions)

If necessary, the framework (stage I) will be further refined to include methods identified from studies in stage II, if these have not already been identified in stage I.

### Stage II: populating the evidence map—identification of evaluations of methods for overviews of systematic reviews of interventions

Inclusion criteria:

i) Systematic reviews of methods studies that have evaluated methods for overviews
ii) Methods studies that have evaluated methods for overviews

Exclusion criteria:

i) Articles published in languages other than English
ii) Methods studies that have evaluated methods for network meta-analysis

In cases where we identify systematic reviews in which the search for studies was conducted before 2013, we will search for more recent methods studies and include the results of these in the evidence map, in addition to including the results of the systematic review.

We plan to populate the evidence map with evaluations of methods that are different or additional to those required to undertake a systematic review of primary research. For example, methods for screening articles for inclusion in an overview (e.g. whether one or more reviewers are required) do not differ to those used in undertaking a systematic review of primary research, so would not be included. However, evaluations of methods used to assess the quality or risk of bias of systematic reviews are of relevance to overviews, and not systematic reviews of primary research, and so would be included. It may be the case that methods have been evaluated in the context of other ‘overview’ products, such as guidelines, and if these methods are of relevance to overviews, they will be included.

The eligibility criteria will be pilot tested by three independent reviewers on a sample of articles retrieved from the search to ensure consistent application.

### Study selection

Citations retrieved from the searches will be imported into EndNote. Duplicates will be identified and removed. Two reviewers will independently review titles and abstracts for their potential inclusion against the selection criteria. Full-text articles will be retrieved when both reviewers agree that inclusion criteria have been met or when there is uncertainty. Two reviewers will then independently assess the articles for inclusion; any disagreement will be resolved by discussion or by arbitration of a third reviewer. In instances where there is limited or incomplete information regarding a study’s eligibility (e.g. when only an abstract is available), the authors will be contacted to request the full text or further details.

### Assessment of the risk of bias of evaluation studies (stage II)

There is no tool available to assess the risk of bias of studies that have evaluated methods (i.e. studies meeting the criteria for stage II). We will therefore report characteristics of the evaluation studies that may plausibly be associated with bias and have been used in other methodology reviews (e.g. [17–19]). These characteristics will include study design, method to select the cohort of studies included in the evaluation, process used to extract data and existence of a protocol. For systematic reviews of methods studies, we will use a tool to assess the risk of bias in systematic reviews (ROBIS) [20].

### Data extraction, data coding and analysis

#### Developing the coding framework

The coding framework will be developed using an iterative process. Two reviewers (CL, SB) will independently code three articles using a simple framework comprised of codes representing the main steps in conducting an overview as outlined in the Cochrane Handbook (e.g. specifying eligibility criteria, data extraction) [21]. Additional codes will be generated inductively by the two reviewers from the text of the three articles. The independently developed frameworks will then be compared and combined into a single integrated framework. The resulting framework will be reviewed by all authors, then tested on three further articles purposively selected to reflect the diversity of article types (i.e. study examining methods in a cohort of overviews, guidance, editorial) included at stage I. Further refinements will be discussed and agreed by all authors. The preliminary framework of parent and illustrative child codes, with descriptions for interpreting each code, is presented in Table 1.

**Table 1:** Coding framework for methods used in overviews of systematic reviews of interventions (stage I)

Parent code	Cues for the coder (description of the code)
Child code	
1. Attributes of the study	Information about the study being coded; includes codes for the purpose and aims and methods of the study.
2. Definition of an overview	Information about the concept and origins of overviews; includes codes for definitions, terminology, rationale and key references for overviews.
3. Terminology for overviews	Description of the terms used for overviews (e.g. ‘review of reviews’). Terms used in the title, abstract and body of the study are coded separately.
4. Methodological considerations (independent of the step or stage of the overview)	Information about the challenges and decisions faced when planning the methods of an overview. Considerations that are unique to overviews should be coded, such as dealing with reviews that (a) overlap in scope, (b) use different methods for assessing bias in included studies, (c) report different outcomes for similar comparisons and (d) have discrepant findings for the same outcomes/comparisons.
4.a. Dealing with currency (or lack of currency) of reviews	Considerations relating to whether a review is up-to-date and the implications of including reviews with older search or publication dates.
4.b. Dealing with reviews of different methodological quality	Considerations relating to the inclusion and interpretation of reviews assessed as being at a high risk of bias (e.g. because of the methods used to identify and select studies, synthesis approach). This may include discussion about exclusion of poorer quality reviews, decisions to weight the findings of reviews based on risk of bias and consideration of risk of bias when grading the evidence arising from a review.
4.c. Dealing with overlap (and differences in scope)	Considerations related to dealing with reviews that include the same studies and overlapping information/data from those studies. Methods relevant to dealing with overlap may apply to one or more steps/stages of the review (e.g. Population, Intervention, Comparison, Outcome (PICO), assessment of quality of included studies, summary and synthesis).
4.d. Dealing with gaps in review coverage	Considerations relating to aspects of the overview question that have not been addressed in reviews (e.g. particular comparisons, types of interventions, subgroups).
4.e. Dealing with missing (or loss of) information about study characteristics	Considerations relating to the information available in reviews about the primary study characteristics (PICO elements, study design). Reviewers report selected information, focusing on that most relevant to their question. This may or may not be congruent with the overview question, and the information may be difficult to interpret out of context, resulting in loss of information.
4.f. Dealing with inconsistent or incomplete outcome reporting across reviews	Considerations when reviews report different outcomes, outcome measures, follow-up times, analyses or data from studies. This may include considerations relating to loss of information if reviews that report unique outcome data are excluded for other reasons (e.g. on the basis of date or quality).
4.g. Dealing with discrepant methods of quality appraisal across reviews	Considerations when reviews use different methods to assess the risk of bias or quality of included studies (e.g. Cochrane Risk of Bias tool [21], Newcastle-Ottawa Quality Assessment Scale [23]).
4.h. Dealing with missing information from the synthesis in reviews	Considerations relating to the type and nature of syntheses (including meta-analyses) reported in the review. Reviewers conduct and report syntheses most pertinent to their question, which may mean syntheses for particular subgroups, interventions or outcomes are not available. Additional considerations may relate to missing information about heterogeneity, sensitivity analyses or other analyses that might be needed to interpret the results of a review.
4.i. Dealing with summary and synthesis of multi-faceted interventions	Considerations relating to the summary and synthesis of findings for multi-faceted interventions. Reviewers may take different approaches to synthesising findings for multi-faceted interventions, driven in part by the question they aim to answer. For example, they might examine potential additive effects of each intervention component or they might decide that interventions are too heterogeneous for meaningful synthesis. Across reviews, this can lead to quite different summaries and syntheses.
4.j. Dealing with discrepant findings across reviews	Considerations when two or more reviews estimate or describe different effects (quantitatively or qualitatively) based on similar studies or data. The findings of two reviews that are similar in scope may differ (a) because their results are different or (b) because the reviewers’ interpretation of the results is different. This node is intended to capture the former.
4.k. Dealing with discrepant interpretation of similar findings across reviews	Considerations when two or more reviews interpret similar results differently. The findings of two reviews that are similar in scope may differ (a) because their results are different or (b) because the reviewers’ interpretation of the results is different. This node is intended to capture the latter.
5. Methods (specific to step or stage of the overview)	Identification, description and other information about the methods used to conduct an overview, grouped by the steps (or stages) described in the Cochrane Handbook [21].
5.a. Overall steps, sequence or stages	Identification or summary of the key steps or stages involved in producing an overview of reviews.
5.b. Specification of scope, questions, objectives	Methods for determining, defining and reporting the scope of an overview; includes factors that influence how broad or narrow questions should be, the ways in which questions might be split (e.g. by condition, by population subgroup, by intervention) and the implications of doing so. This node relates to the specification of eligibility criteria but differs in that it covers conceptual issues and the methodological implications of lumping/splitting.
5.c. Specification of eligibility criteria	Methods for determining and specifying eligibility criteria (i.e. PICO); includes restrictions on eligibility (e.g. publication status, year) that may be used to deal with overlap; includes codes for outcome selection.
5.c.i. Outcome selection mechanisms	Methods for specification and selection of outcomes. Note that while information about outcome selection methods can be reported under eligibility criteria, reviews are not necessarily excluded from the overview if they do not report a specified outcome. Instead, the outcomes reported may determine inclusion in the synthesis (or summary) of effects. In circumstances where reviews are included irrespective of outcomes, information relating to outcome specification may need to be coded under other relevant nodes (e.g. ‘Outcome selection mechanisms’).
5.c.ii. Decision rules for selecting a review from multiple overlapping reviews	Methods for dealing with multiple reviews that include the same studies and overlapping data from those studies. Several methods have been proposed to deal with overlap and are represented in the subnodes. These include (a) the review with the largest amount of studies, (b) most recent/up-to-date information, (c) the highest quality studies, (d) most complete reporting, (e) by publication status, (f) one review per author, (g) eliminate the review with the least amount of studies and (h) ignore overlap.
5.d. Search methods	Methods for searching for reviews in an overview; includes codes for specific search filters used, such as those provided in PubMed.
5.e. Selection of reviews	Methods for selecting reviews for inclusion in the overview, such as the use of two reviewers to independently screen reviews for inclusion in the overview.
5.f. Data extraction and coding	Methods for data extraction in an overview and coding of information from the included reviews; includes codes for extracting data for subgroups, extracting data from reviews versus trials, and methods used to select outcomes for extraction.
5.g. Assessment of risk of bias (methodological quality) of primary studies included in the reviews	Methods for dealing with reviews that have used different methods or tools for assessing risk of bias (or quality) of primary studies.
5.g. Assessment of risk of bias arising from the methods of the review	Methods for assessing the risk of bias arising from the design, conduct and reporting of reviews. Sometimes referred to as methodological quality of the review. Example tools include ROBIS [20], A Measurement Tool to Assess Systematic Reviews (AMSTAR) [24], National Institute for Health and Care Excellence (NICE) [25].
5.g. Assessment of the overall quality of the evidence arising from the overview	Methods for assessing the overall quality of the evidence for each comparison/outcome in the overview. These methods are likely to be the same as used for any synthesis (e.g. Grades of Recommendation, Assessment, Development and Evaluation (GRADE) [26], Scottish Intercollegiate Guidelines Network (SIGN) [27], FORM [28]) but may require adaptation to synthesise evidence across reviews instead of primary studies. For example, should the assessment of risk of bias in the included review be considered when grading the evidence and, if so, how?
5.h. Synthesis	Methods for analysing and synthesising the data in an overview; includes approaches for dealing with meta-analyses with overlapping studies from different reviews (i.e. those for the same comparison and outcomes), exploring heterogeneity, etc.
5.h.i. Quantitative synthesis	Methods for quantitative synthesis in an overview; includes codes for synthesising meta-analyses in an overview, exploring heterogeneity among included reviews, considerations of how to include meta-analyses and how to include subgroup analyses and synthesis and summary without meta-analysis.
5.h.ii. Synthesis and summary without meta-analysis	Methods for synthesis and summary that do not include a meta-analysis (e.g. plotting and tabulating data, vote counting).
5.i. Presentation and summary of findings	Methods for presenting and summarising the findings of an overview. May include methods for efficient depiction of the overlap of studies across included reviews, methods for summarising findings when the same study is included in more than one review, etc.
5.j. Interpreting findings and drawing conclusions	Methods for interpreting results and drawing conclusions in an overview.
5.k. Reporting	Recommendations about the preferred reporting items for overviews, which may involve using standards or guidelines for reporting overviews, checklists or reporting tools.
6. Pros and cons of method	Known or anticipated benefits and disadvantages of using different methods in an overview. These might relate to efficiency of production, utility of the overview for decision makers and the validity of findings (bias in estimates of intervention effects).

#### Stage I: populating the framework

Data will be coded from articles meeting the inclusion criteria of stage I using NVivo software. Coded data will be analysed thematically to further refine and populate the framework with descriptions of each method. This thematic analysis will be used to (1) determine the most appropriate categorisation of methods (i.e. the final structure and terminology used in the framework); (2) determine whether or not methods are distinct (i.e. identifying whether methods described using different terminology are indeed different and vice versa); and (3) write a description of each method by synthesising key concepts across included studies. In addition, data will be coded to identify the type of paper (article describing method for overviews, cohort/cross-sectional; commentary or editorial; guidance document); noted advantages/disadvantages of the method; examples of use; at which step in the overview process the method is used; and unique methodological considerations in conducting overviews.

#### Stage II: populating the evidence map

We plan to extract the following data from methods evaluation studies using a data extraction form: characteristics of the article (e.g. publication year, journal); characteristics of the study (study design, type of sample selection (e.g. random, consecutive, others), process to extract data, existence of a protocol); primary objective(s); secondary objective(s); methods evaluated for primary objective; evaluation analysis methods; and the quantitative results relating to the primary objective.

Coding and data extraction for both stages of the study will be undertaken independently by two reviewers. We anticipate that it will be difficult to pre-specify response categories for each data extraction item due to the potential variability in the type of evaluations. Therefore, much of the extracted data will be verbatim free text, which will be categorised following discussion between the reviewers. Discrepancies in extracted data will be discussed between the reviewers until consensus is reached or by arbitration of a third reviewer if required.

### Data analysis

We will describe the evaluations that have been undertaken and map these evaluations to the framework of methods identified in stage I. The description of evaluations will include the yield and characteristics of the available evidence, the use, advantages and disadvantages of each method and a summary of the findings of the evaluations.

## Discussion

Methods for conducting, interpreting and reporting overviews are in their infancy. To date, there has been no systematic review or evidence map examining either the range of methods for overviews or the evidence behind those methods. We plan to use evidence mapping methods to develop and populate a framework of methods used in conducting, interpreting and reporting overviews of systematic reviews of interventions (stage I) and create an evidence map of studies that have evaluated these methods (stage II). This will be a novel application of the evidence mapping methodology in a context where, to our knowledge, this method has not been used.

The results of the evidence map may influence the use of particular methods in overviews, either directly or by influencing guidance for overview methods. However, perhaps the most notable use of the evidence map will be in directing methods research to areas where there is limited evidence for the methods that are being used. Such a map may also be useful for national funding agencies in considering what methods research should be funded.

### Strengths and limitations

We have developed a protocol to guide our research and reduce post hoc decision making. Two reviewers will undertake the screening, data coding and data extraction of all methods studies. Searching for methodological papers can prove very difficult in databases other than specialist methodology collections, such as the Cochrane Methodology Register and the Meth4ReSyn library [22]. In addition, while the coverage of two of the specialist methodology collections extends beyond the health and medical literature (Meth4ReSyn and Scientific Resource Center Methods library), the focus of our search is within health. These factors might mean that some relevant methodology papers are missed. However, we have included reference checking, citation searching and contacting relevant experts in the field to minimise the impact of these limitations.

### Research status

At the time of submitting this protocol, we have undertaken the searching and screening for stage I. Two reviewers have independently coded three methods papers for stage I and developed an initial framework (Table 1). During the peer-review process, we amended the inclusion criteria to clarify that our focus was on methods for overviews of systematic reviews of interventions. We also revised our search strategies in response to suggestions from the reviewer.

### Summary

Our results will lead to an inventory of evaluation studies of methods for overviews of systematic reviews of interventions. The evidence map will aid in the development and implementation of methods for overviews which will be relevant to a wide range of knowledge users, including researchers, funders and journal editors. The evidence map will help to prioritise the future research agenda in this field.

## Additional files

Additional file 1:
**PRISMA-P: recommended items to address in a systematic review protocol.** The Preferred Reporting Items for Systematic reviews and Meta-Analyses Protocols (PRISMA-P) [14] was used to develop this protocol. The PRISMA-P checklist was developed for the preparation of protocols of systematic reviews that summarise aggregate data from studies, particularly the evaluations of the effects of interventions, and therefore, not all PRISMA-P items were applicable. (DOC 54 kb) Additional file 2:
**Search strategies.** The search strategies that will be used to identify eligible studies. (DOC 24 kb)

## Abbreviations

PICO: Population, Intervention, Comparison and Outcomes
PRISMA-P: Preferred Reporting Items for Systematic Reviews and Meta-Analyses for Protocols
ROBIS: a tool for assessing the risk of bias in systematic reviews
