# Supplementary material for: Evidence map of studies evaluating methods for conducting, interpreting and reporting overviews of systematic reviews of interventions: rationale and design
Source: Syst Rev. 2016 Jan 6;5:4. doi: 10.1186/s13643-015-0178-0 (PMC4702312; doi:10.1186/s13643-015-0178-0)
Supplement: Additional file 2: — Search strategies. The search strategies that will be used to identify eligible studies. (DOC 24 kb) [file 13643_2015_178_MOESM2_ESM.doc]

**Additional file 2 - Search strategies**

Cochrane Methodology Register

(www.cochranelibrary.com)

"overviews" or (overview* and review*) or metareview* or meta-review* or meta-synthesis or meta-syntheses or umbrella or "Cochrane overview*" or “review* of reviews” or "review* of systematic reviews"

Meth4ReSyn library

(www.citeulike.org/user/Meth4ReSyn)

overviews or (overview && reviews) or metareviews or “meta-reviews” or “meta-synthesis” or “meta-syntheses” or umbrella or “Cochrane overview” or “review of reviews” or “reviews of reviews” or “review of systematic reviews” or “reviews of systematic reviews” or (tag: review_of_reviews)

Cochrane Colloquium abstracts

(http://abstracts.cochrane.org)

"overviews" or (overview* and review*) or metareview* or meta-review* or meta-synthesis or meta-syntheses or umbrella or "Cochrane overview*" or "review* of reviews" or "review* of systematic reviews"

Scientific Resource Center Methods library of the AHRQ Effective Health Care Program

(http://www.citeulike.org/user/SRCMethodsLibrary)

overviews or (overview && reviews) or metareviews or “meta-reviews” or “meta-synthesis” or “meta-syntheses” or umbrella or “Cochrane overview” or “review of reviews” or “reviews of reviews” or “review of systematic reviews” or “reviews of systematic reviews” or (tag: systematic-reviews---reviews of) or (tag: systematic-reviews)

**Ovid MEDLINE**

((overview$ or review) and reviews).ti. or "umbrella reviews".ti,ab. or (meta-reviews or metareviews).ti,ab. or metasyntheses.ti,ab. or "reviews of systematic reviews".ti,ab. or "reviews of reviews".ti,ab. or (overviews adj4 reviews).ab.
